# The Association of Screen Time, Sleep Quality, and Dry Eye Among College Students in Saudi Arabia

**DOI:** 10.7759/cureus.37533

**Published:** 2023-04-13

**Authors:** Eman D Albalawi, Sarah K Alswayed, Sarah S Aldharman, Asmaa Y Alshangiti, Ghadah A Alhussein, Halah O Alamawi

**Affiliations:** 1 Department of Ophthalmology, Princess Nourah bint Abdulrahman University, Riyadh, SAU; 2 College of Medicine, King Saud Bin Abdulaziz University for Health Sciences, Riyadh, SAU; 3 College of Medicine, Princess Nourah bint Abdulrahman University, Riyadh, SAU

**Keywords:** saudi arabia, college students, sleep difficulties, screen time, dry eye

## Abstract

Background

Dry eye is a serious public health issue that causes ocular discomfort, weariness, and visual disturbances that can disrupt everyday activities. Dry eye disease is one of the most common reasons people seek eye care. Therefore, this study aimed to assess the association between screen time, sleep quality, and dry eye among college students in Saudi Arabia.

Methods

This cross-sectional study was conducted among college students in Saudi Arabia. Data were collected through a validated questionnaire distributed via social media.

Results

A total of 1,593 participants were included. Many of them were aged between 18-25 years (80.7%) and females were (65.0%). Females and residents of the middle region had significantly more severe sleep-wake difficulties than other people (p<0.001). Participants with a master's degree had lower severe sleep-wake difficulties than other participants (p<0.001). Participants who spent between 4-6 hours on the screen showed high severe sleep-wake difficulties (p<0.001). Regarding eye dryness, females, participants with a bachelor's degree, and participants who spent more than six hours on screen had more severe symptoms of eye dryness. Nearly half of the participants with severe sleep-wake difficulties reported mild to moderate symptoms of dry eye (p<0.001).

Conclusions

Our study concluded that university students in Saudi Arabia had significant sleep-cycle difficulties and mild to moderate eye dryness symptoms. Age, female gender, sleep duration, educational level, monthly income, and excessive screen time were found to be associated with sleep-cycle problems and eye dryness symptoms.

## Introduction

Computer vision syndrome (CVS) is a recognized health concern that is characterized by a variety of eye and vision-related symptoms [1]. The prevalence of computer vision syndrome is greater than 50% among computer users [1]. The emergence of more websites and social networks has persuaded young people to spend more time staring at electronic devices or computer screens. There has been a wide increase in online educational resources and amusement platforms for video games and movies in the last 10 years. Therefore, young people's time spent on screen has increased steadily in many nations [1]. A relationship between greater multimedia exposure and health problems has been identified in several previous pieces of research [1]. Although the negative effects of smartphone radiation are widely understood, the public is less aware of the extra implications the increased screen time has on well-being, including disruptions in the circadian cycle and stress on the musculoskeletal system, as well as the visual system.

Digital devices produce electromagnetic fields and blue light emissions that cause disruptions to the circadian rhythm [2]. Blue light suppresses melatonin, which is a sleep promoter [3,4]. There are two types of computer symptoms: those connected with accommodation (e.g., blurring of vision when focusing) and those associated with dry eyes (e.g., burning and grittiness) [5]. Millions of people throughout the world suffer from dry eye disease (DED), with prevalence rates in different communities ranging from 5% to 50% [6]. The ocular symptoms of DED include pain, hazy vision, sensitivity to light, and ocular burning [6].

DED is a multifactorial condition of the ocular surface that is caused by alterations in the tear matrix [7]. DED is divided into two categories: episodic and chronic. Of the two, episodic DED is caused by a variety of factors, such as extended visual tasks with decreased blinking, and it advances to chronic DED when these symptoms persist [7]. Given the increasing prevalence of dry eye disease (DED) among the Saudi population, which accounts for 32.1% of the population, it is important to examine the potential risk factors [7]. The severity of DED may significantly affect the quality of life (QOL) in different life aspects. Visual discomfort may interfere with daily tasks, including reading and driving, as well as limited involvement in work and social life [8-11].

Furthermore, work productivity is significantly correlated with the severity of DED [12]. In a large study conducted in Saudi Arabia on the productivity of the worker, the DED contributes to low productivity in different ways, either by work limitation, impaired work performance, or absence from work [12]. Moreover, DED is associated with a significantly increased economic burden. One DED patient's average annual direct costs for ophthalmologist-managed care are estimated to be $800 in the US. In addition, the financial cost increases profoundly when lost productivity at work is considered. In the US, DED's annual direct healthcare costs are around $4 billion, while its annual productivity losses might approach $55 billion [13].

Various risk factors are associated with DED, including age, female gender, contact lens wearer, smoking, low humidity, screen time, and sleep quality. Time spent on digital screens is increasingly considered one of the most relevant risk factors of DED [14]. Interestingly, several studies have demonstrated the effect of blinking dynamics as a contributing factor to vision-related problems among digital screen users [14]. Moreover, several previous studies have found that poor sleep quality can lead to an increase in tear osmolarity and a decrease in tear production, which explains the link between poor sleep quality and DED [7].

Despite the size of the literature on the association between dry eye disease, sleep quality, and screen time separately, limited data are available to shed light on the DED association between sleep quality and digital screen use in one study. Therefore, this study aimed to investigate the association between dry eye, sleep quality, and digital screen time among university students in Saudi Arabia.

## Materials and methods

A descriptive cross-sectional questionnaire-based study was conducted in Saudi Arabia between September 2022 and November 2022. The target population was college students from different regions (Central, Southern, Eastern, Western, and Northern). The data were gathered via a self-administered questionnaire. The questionnaire was distributed electronically via Google Forms (Google LLC, Mountain View, CA). The data were entered into Microsoft Excel (Microsoft Corp., Redmond, WA) and then analyzed employing the Statistical Package for the Social Sciences (SPSS) software (IBM Corp., Armonk, NY).

Inclusion criteria and exclusion criteria

College students from different regions in Saudi Arabia were included in this study. Participants who did not fill out the whole questionnaire or did not agree to participate were excluded from the study.

Sampling technique and sample size calculation

OpenEpi® version 3.0 software (www.OpenEpi.com) was used to estimate the sample size. The representative sample size needed is 385, with a margin error of 5% and a confidence level of 95%. We intended to get more than the estimated sample size to overcome any exclusions. The non-probability consecutive sampling technique was used.

Data collection instrument and procedure

A pre-validated and structured questionnaire, consisting of four sections, was used. The first section included information on the participants' socioeconomic and demographic characteristics. The second section focused on screen time and usage such as the daily hours spent on screen, the device used the most, and the main purpose for using the screen. The third section was related to the sleep-wake cycle using the Mini Sleep Questionnaire framework, which contains 10 items using a seven-point Likert scale [15]. It covered the following: difficulty falling asleep, waking up too early, hypnotic medication use, falling asleep during the day, feeling tired upon waking up in the morning, snoring, mid‐sleep awakenings, headaches on awakening, excessive daytime sleepiness, excessive movement during sleep. The last section emphasized dry eye symptoms using the Standard Patient Evaluation of Eye Dryness (SPEED), which is centered on the occurrence, frequency, and severity of the following symptoms: dryness, grittiness, or scratchiness, soreness or irritation, burning or watering, and eye fatigue [16]. The questionnaire was translated into the Arabic language by an expert to make it easier for the public to read and understand. The survey findings were then translated into English using certified translation tools.

An electronic Google form of the questionnaire was distributed on different social media platforms such as WhatsApp, Twitter, and Telegram. All information was kept private and was only used for scientific research. Participation in this study was entirely voluntary and optional with informed consent provided to all participants on the first page before filling out the questionnaire. Ethical approval was obtained from the institutional review board at Princess Nourah bint Abdulrahman University (Reference number: 22-0405).

Data management and statistical analysis

After finishing filling out the surveys, they were checked for completeness and any missing information. Then, the data were entered into Microsoft Excel. After data were extracted, it was revised, coded, and fed to statistical software IBM SPSS version 22 (SPSS, Inc. Chicago, IL). The mean ± standard deviation (SD) was reported for continuous variables while categorical variables were described using frequencies and percentages. Analysis of quantitative data by one-way analysis of variance (ANOVA) test and association of qualitative variables by a chi-square test was conducted. A p-value <0.05 was considered significant.

## Results

Characteristics of the participants

A total of 1716 respondents filled out the questionnaire. After applying the exclusion criteria, 1593 participants were included in the final analysis of the study. The most common age group was 18-25 years (80.7%) followed by 26-35 years (16.3%) and only (3%) were in the age of 36-45 years. Furthermore, over half the participants were females (65%). The majority of respondents were single (80.9%) and had a bachelor’s degree (85.4%). Also, the distribution of participants’ residences was different across Saudi Arabia. Most of them were from the central region (26.6%) and only a minority (14.9%) were from the southern region. Most of the participants have an average monthly income of 5000-10,000 SAR (31.3%) or 10,000-20,000 SAR (31.2%). Regarding the number of hours they spent in front of the screen, nearly half of them spent more than six hours (47.2%). Further information is presented in Table 1.

**Table 1:** Sociodemographic characteristics of the study participants

Variable	Characteristic	Overall, N = 1,593
Age (in years)	18-25	1,286 (80.7%)
	26-35	259 (16.3%)
	36-45	48 (3%)
Gender	Female	1,035 (65%)
	Male	558 (35%)
Marital status	Single	1,289 (80.9%)
	Married	252 (15.8%)
	Divorced	44 (2.8%)
	Widow	8 (0.5%)
Educational level	Diploma	94 (5.9%)
	Bachelor’s degree	1,360 (85.4%)
	Master’s degree	118 (7.4%)
	Doctorate degree	21 (1.3%)
Which part of Saudi Arabia do you reside in?	Eastern region	264 (16.6%)
	Middle region	424 (26.6%)
	Northern region	348 (21.8%)
	Southern region	238 (14.9%)
	Western region	319 (20%)
What is your monthly home income (SAR)?	<5000	209 (13.1%)
	5000-10,000	499 (31.3%)
	10,000-20,000	497 (31.2%)
	> 20,000	388 (24.4%)
No. of hours daily time (the number of hours spent in front of the screen)?	0–2 h	96 (6.0%)
	2–4 h	329 (20.7%)
	4-6 h	416 (26.1%)
	More than 6 h	752 (47.2%)
Device on which maximum time is spent	Laptop/Desktop	253 (15.9%)
	Mobile Phone	915 (57.4%)
	Tablet/iPad	384 (24.1%)
	Television	41 (2.6%)
Purpose	Social media	1,212 (76.1%)
	Studies	1,181 (74.1%)
	Movies	655 (41.1%)
	Gaming	374 (23.5%)

Sleep quality

**Figure 1:**
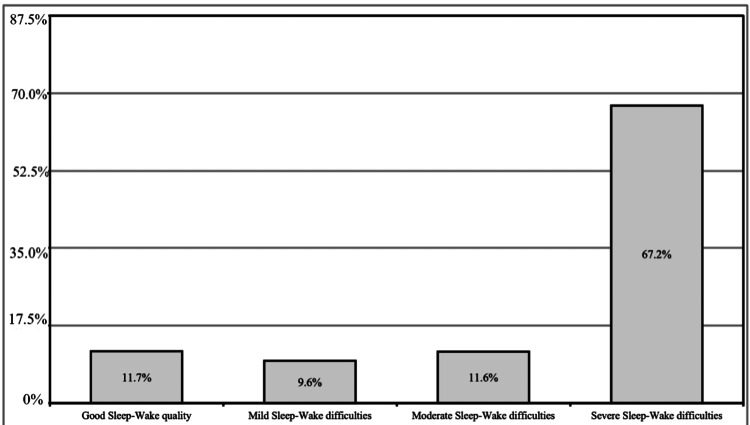
Sleep-wake quality

In this study, we assessed the distribution of sleep-wake, and it was found that most of the participants had severe sleep-wake difficulties (n=1,070, 67.2%), 184 (11.6%) had moderate sleep-wake difficulties, and 153 (9.6%) had mild sleep-wake difficulties while only 186 (11.7%) of the participants had good sleep-wake quality as shown in Figure 1.

Regarding the association between sleep-wake quality and different sociodemographic characteristics, we found that there were significant associations between age, gender, marital status, education, residence, and number of hours daily time in relation to sleep-wake quality (P value = 0.001, < 0.001, 0.028, 0.018, < 0.001, < 0.001, respectively). The age group of 26-35 years, female gender and single, and married participants showed high severe sleep-wake difficulties. For residence, the population from the middle region had more severe sleep-wake difficulties than other people. Educational level showed that participants with diplomas had higher severe sleep-wake difficulties than other participants. Regarding the number of hours daily time, participants who spent between four and six hours or more than six hours in front of a screen showed high severe sleep-wake difficulties as compared to others. The only exception without significant difference was monthly home income (P = 0.298). Table 2 shows the association between sleep-wake and sociodemographic characteristics.

**Table 2:** Association between sleep-wake and sociodemographic characteristics *: statistically significant

Characteristic	Sleep-wake				P value
	Good Sleep-Wake quality, N = 186	Mild Sleep-Wake difficulties, N = 153	Moderate Sleep-Wake difficulties, N = 184	Severe Sleep-Wake difficulties, N = 1,070	
Age (in years)					
18-25	143 (11.1%)	126 (9.8%)	158 (12.3%)	859 (66.8%)	0.001*
26-35	35 (13.5%)	25 (9.7%)	14 (5.4%)	185 (71.4%)	
36-45	8 (16.7%)	2 (4.2%)	12 (25%)	26 (54.2%)	
Gender					
Female	92 (8.9%)	93 (9%)	117 (11.3%)	733 (70.8%)	< 0.001*
Male	94 (16.8%)	60 (10.8%)	67 (12%)	337 (60.4%)	
Marital status					
Single	142 (11%)	123 (9.5%)	151 (11.7%)	873 (67.7%)	0.028*
Married	33 (13.1%)	21 (8.3%)	28 (11.1%)	170 (67.5%)	
Divorced	8 (18.2%)	8 (18.2%)	3 (6.8%)	25 (56.8%)	
Widow	3 (37.5%)	1 (12.5%)	2 (25%)	2 (25%)	
Educational level					
Diploma	5 (5.3%)	4 (4.3%)	11 (11.7%)	74 (78.7%)	0.018*
Bachelor’s degree	155 (11.4%)	132 (9.7%)	157 (11.5%)	916 (67.4%)	
Master’s degree	24 (20.3%)	13 (11%)	13 (11%)	68 (57.6%)	
Doctorate degree	2 (9.5%)	4 (19%)	3 (14.3%)	12 (57.1%)	
Which part of Saudi Arabia do you reside in?					
Eastern region	30 (11.4%)	25 (9.5%)	36 (13.6%)	173 (65.5%)	< 0.001*
Middle region	36 (8.5%)	29 (6.8%)	42 (9.9%)	317 (74.8%)	
Northern region	60 (17.2%)	34 (9.8%)	47 (13.5%)	207 (59.5%)	
Southern region	18 (7.6%)	25 (10.5%)	23 (9.7%)	172 (72.3%)	
Western region	42 (13.2%)	40 (12.5%)	36 (11.3%)	201 (63%)	
What is your monthly home income (SAR)?					
<5000	25 (12%)	19 (9.1%)	32 (15.3%)	133 (63.6%)	0.298
5000-10,000	60 (12%)	39 (7.8%)	47 (9.4%)	353 (70.7%)	
10,000-20,000	58 (11.7%)	51 (10.3%)	54 (10.9%)	334 (67.2%)	
> 20,000	43 (11.1%)	44 (11.3%)	51 (13.1%)	250 (64.4%)	
No. of hours daily time (Q. The number of hours spent in front of the screen)					
0–2 h	25 (26%)	12 (12.5%)	14 (14.6%)	45 (46.9%)	< 0.001*
2–4 h	56 (17%)	27 (8.2%)	35 (10.6%)	211 (64.1%)	
4-6 h	37 (8.9%)	42 (10.1%)	41 (9.9%)	296 (71.2%)	
More than 6 h	68 (9%)	72 (9.6%)	94 (12.5%)	518 (68.9%)	

Eye dryness

**Figure 2:**
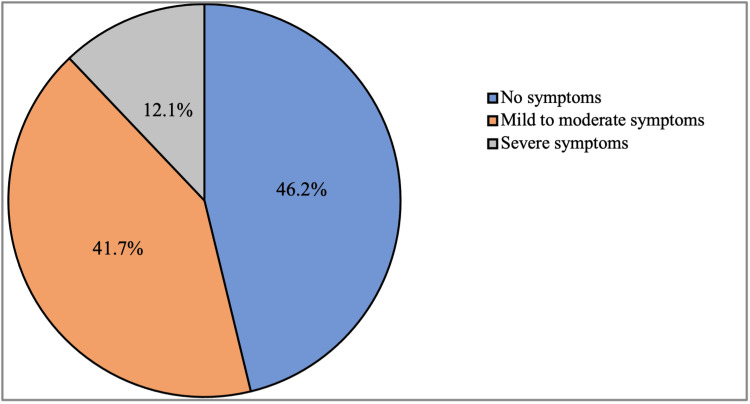
Severity of eye dryness symptoms

Our results revealed that the overall eye dryness score was 7.2 ± 5.9 (range 0 - 28), and most of the participants had no symptoms of eye dryness (46.2%). About 41.7% experienced mild to moderated symptoms of eye dryness, and the least percentage of them showed severe symptoms of eye dryness (12.1%) as shown in Figure 2.

Regarding the association of different variables with eye dryness; our results found that age was significantly associated with eye dryness. Participants belonging to the 18-25 and 36-45 age groups were significantly associated with higher severe symptoms than other age groups (P < 0.001). Moreover, we found that female gender and single participants were associated with higher severe symptoms than others, and these differences came to be significant (P < 0.001).

Regarding educational level, respondents with a doctorate degree were significantly associated with a higher percentage of severe symptoms of eye dryness (P < 0.001). In addition, we reported that there was a significant association between eye dryness and monthly income, participants who had more than 20,000 SAR monthly income had more severe symptoms than others (P = 0.003). Furthermore, the duration of hours spent in front of the screen was significantly associated with eye dryness (P < 0.001), as we found that respondents who spent more than six hours duration had the highest percentage of severe symptoms. We also found that there was no significant association between residence and eye dryness (P = 0.072). Details on the association of different variables with eye dryness are shown in Table 3.

**Table 3:** Association of different variables with eye dryness *: statistically significant

Characteristic	Eye dryness			P value
	No symptoms, N = 736	Mild to moderate symptoms, N = 664	Severe symptoms, N = 193	
Age				
18-25	553 (43%)	560 (43.5%)	173 (13.5%)	< 0.001*
26-35	161 (62.2%)	84 (32.4%)	14 (5.4%)	
36-45	22 (45.8%)	20 (41.7%)	6 (12.5%)	
Gender				
Female	399 (38.6%)	467 (45.1%)	169 (16.3%)	< 0.001*
Male	197 (35.3%)	337 (60.4%)	24 (4.3%)	
Marital status				
Single	555 (43.1%)	559 (43.4%)	175 (13.6%)	< 0.001*
Married	156 (61.9%)	81 (32.1%)	15 (6%)	
Divorced	20 (45.5%)	21 (47.7%)	3 (6.8%)	
Widow	5 (62.5%)	3 (37.5%)	0 (0%)	
Educational level				
Diploma	50 (53.2%)	40 (42.6%)	4 (4.3%)	< 0.001*
Bachelor’s degree	602 (44.3%)	578 (42.5%)	180 (13.2%)	
Master’s degree	74 (62.7%)	38 (32.2%)	6 (5.1%)	
Doctorate degree	10 (47.6%)	8 (38.1%)	3 (14.3%)	
Which part of Saudi Arabia do you reside in?				
Eastern region	119 (45.1%)	106 (40.2%)	39 (14.8%)	0.072
Middle region	198 (46.7%)	175 (41.3%)	51 (12%)	
Northern region	149 (42.8%)	148 (42.5%)	51 (14.7%)	
Southern region	129 (54.2%)	91 (38.2%)	18 (7.6%)	
Western region	141 (44.2%)	144 (45.1%)	34 (10.7%)	
What is monthly home income (SAR)?				
<5000	104 (49.8%)	77 (36.8%)	28 (13.4%)	0.003*
5000-10,000	250 (50.1%)	198 (39.7%)	51 (10.2%)	
10,000-20,000	236 (47.5%)	209 (42.1%)	52 (10.5%)	
> 20,000	146 (37.6%)	180 (46.4%)	62 (16%)	
No. of hours daily time (Q. The number of hours spent in front of the screen)				
0–2 h	67 (69.8%)	24 (25%)	5 (5.2%)	< 0.001*
2–4 h	228 (69.3%)	87 (26.4%)	14 (4.3%)	
4-6 h	180 (43.3%)	198 (47.6%)	38 (9.1%)	
More than 6 h	261 (34.7%)	355 (47.2%)	136 (18.1%)	

Our results showed that there was a significant association between sleep-wake and the device on which maximum time was spent, as we showed below participants who used Tablet/iPad had more severe sleep-wake difficulties than others (70.6%) (P < 0.001) and participants who used mobile phone or television experienced moderate sleep-wake issues compared to others. In regards to the purpose of using the device, we found that respondents who used the device for studying had more percentage of severe sleep-wake difficulties than others, and this difference was found to be significant (67.9%) (P < 0.001). Moreover, there is a significant association between sleep-wake and watching movies (P = 0.029).

Our findings revealed that participants with severe sleep-wake difficulties were significantly associated with the highest eye dryness score of 8.1 ± 5.9, whereas respondents who had good sleep-wake difficulties were associated with the lowest eye dryness score of 4.0 ± 5.1 (P < 0.001). There is a significant association between sleep-wake difficulties and eye dryness. Participants who had no symptoms were significantly associated with good sleep-wake quality (P < 0.001). Lastly, eye fatigue showed a significant association with sleep-wake difficulties (P < 0.001) as demonstrated in Table 4.

**Table 4:** Association between sleep-wake quality and used devices, purpose of use, and eye dryness *: statistically significant

Characteristic	Sleep-wake				P value
	Good SleepWake quality, N = 186	Mild SleepWake difficulties, N = 153	Moderate SleepWake difficulties, N = 184	Severe SleepWake difficulties, N = 1,070	
Device on which maximum time is spent					
Laptop/Desktop	38 (15%)	26 (10.3%)	21 (8.3%)	168 (66.4%)	<0.001*
Mobile Phone	108 (11.8%)	76 (8.3%)	120 (13.1%)	611 (66.8%)	
Tablet/iPad	30 (7.8%)	46 (12%)	37 (9.6%)	271 (70.6%)	
Television	10 (24.4%)	5 (12.2%)	6 (14.6%)	20 (48.8%)	
Purpose					
Social media	133 (11%)	114 (9.4%)	143 (11.8%)	822 (67.8%)	0.4
Studies	115 (9.7%)	125 (20.6%)	139 (11.8%)	802 (67.9%)	<0.001*
Movies	81 (12.4%)	70 (10.7%)	90 (13.7%)	414 (63.2%)	0.029*
Gaming	41 (11%)	37 (9.9%)	51 (13.6%)	245 (65.5%)	0.5
Eye dryness score	4.0 ± 5.1	5.5 ± 5.4	6.3 ± 5.4	8.1 ± 5.9	<0.001*
Eye dryness					
No symptoms	134 (18.2%)	90 (12.2%)	98 (13.3%)	414 (56.3%)	<0.001*
Mild to moderate symptoms	44 (6.6%)	54 (8.1%)	68 (10.2%)	498 (75%)	
Severe symptoms	8 (4.1%)	9 (4.7%)	18 (9.3%)	158 (81.9%)	
Eye fatigue	64 (34.4%)	75 (49.0%)	110 (59.8%)	695 (65.0%)	<0.001*

As shown in Table 5, there was a significant correlation between eye dryness and the device on which participants spent most of their time (P < 0.001), participants who used a Tablet/iPad experienced more severe eye dryness symptoms than other participants (18.2%). In terms of the device's intended use, we discovered that respondents who used it for academic purposes had a higher percentage of severe symptoms of eye dryness than other respondents (14.5%), and this difference was significant (P < 0.001). Additionally, there is a significant association between eye dryness and watching movies (P = 0.005).

According to our research, participants who experienced severe eye dryness symptoms substantially had the greatest eye sleep-wake score of 39.5 ± 9.2, whereas those who did not experience any eye dryness symptoms had the lowest sleep-wake score of 31.4 ± 8.1 (P < 0.001). Eye dryness and sleep-wake issues are significantly correlated. Participants who did not experience any symptoms had better sleep-wake quality (P < 0.001). Our research revealed a statistically significant relationship between eye fatigue and eye dryness (P < 0.001).

**Table 5:** Association between eye dryness and used devices, purpose of use, and sleep-wake quality *: statistically significant

Characteristic	Eye dryness			P value
	No symptoms, N = 736	Mild to moderate symptoms, N = 664	Severe symptoms, N = 193	
Device on which maximum time was spent				
Laptop/Desktop	147 (58.1%)	82 (32.4%)	24 (9.5%)	< 0.001*
Mobile Phone	441 (48.2%)	378 (41.3%)	96 (10.5%)	
Tablet/iPad	125 (32.6%)	189 (49.2%)	70 (18.2%)	
Television	23 (56.1%)	15 (36.6%)	3 (7.3%)	
Purpose				
Social media	567 (46.8%)	508 (41.9%)	137 (11.3%)	0.2
Studies	484 (41%)	526 (44.5%)	171 (14.5%)	< 0.001*
Movies	272 (41.5%)	302 (46.1%)	81 (12.4%)	0.005*
Gaming	160 (42.8%)	172 (46%)	42 (11.2%)	0.2
Sleep-wake score	31.4 ± 8.1	35.8 ± 8.2	39.5 ± 9.2	< 0.001*
Overall sleep-wake score	34.2 ± 8.8			
Sleep-wake				
Good sleep-wake quality	134 (72%)	44 (23.7%)	8 (4.3%)	< 0.001*
Mild sleep-wake difficulties	90 (58.8%)	54 (35.3%)	9 (5.9%)	
Moderate sleep-wake difficulties	98 (53.3%)	68 (37%)	18 (9.8%)	
Severe sleep-wake difficulties	414 (38.7%)	498 (46.5%)	158 (14.8%)	
Eye fatigue	243 (33.0%)	526 (79.2%)	175 (90.7%)	< 0.001*
Eye dryness score	9.5 ± 2.5	2.1 ± 1.9	18.3 ± 3.5	<0.001*
Overall eye dryness score	7.2 ± 5.9			

**Figure 3:**
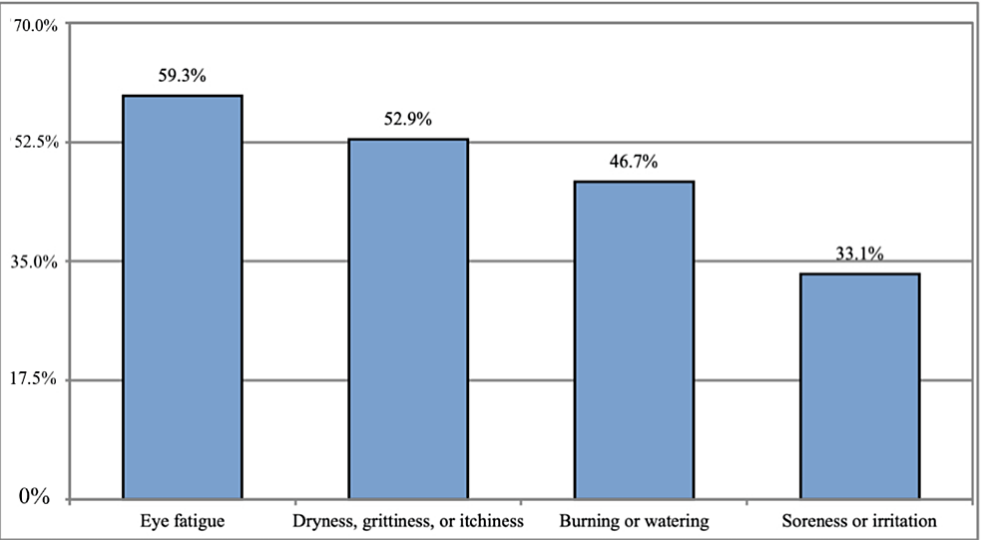
Types of symptoms experienced by participants within the last three months

Our findings reported that eye fatigue was the most experienced symptom by participants during the last three months (59.3%) followed by dryness, grittiness or itching (53.9%), burning or watering (46.7%), and soreness or irritation (33.1%) as demonstrated on Figure 3.

## Discussion

Dry eye (DE) is a multifactorial condition of the tears and the ocular surface that causes discomfort, visual disruption, and tear film instability, as well as potential ocular surface injury. It is accompanied by increased tear film osmolarity and ocular surface irritation [17]. The current study aimed to assess the association between screen time, sleep quality, and dry eye among college students in Saudi Arabia.

Our findings revealed that participants aged 18-25 years were considerably more likely to have severe sleep-wake issues than other age groups. Similarly, a study that was conducted in Spain found that participants aged from 18 to 21 years had severe sleep-wake difficulties [18]. Furthermore, our data showed that the female gender was correlated with significant sleep-waking issues (p=0.001). This could be because females made up the majority of our study population. Our findings showed that participants who spent four to six hours on screen had severe sleep-cycle difficulties (p0.001). As the body delays melatonin generation when using smartphones at night, the circadian rhythm is disrupted [19].

Regarding factors associated with eye dryness, participants aged 26-35 years in our study had more severe symptoms than other age groups (p<0.001). This is in contrast to a study that was conducted in Indonesia, which showed that dry eye symptoms increased with age [20]. The function of the lacrimal gland has been shown to steadily decline with age, resulting in decreased tear output and eye dryness illness in the elderly [21]. Moreover, a prior study in Japan found an age-related trend in the prevalence of severe dry eye disease symptoms exclusively in women [22]. In our study, we found that females suffered more from severe dry eye symptoms than males (p<0.001). This was inconsistent with another study, which showed that dry eye symptoms increased among the male gender [20]. However, a Korean study confirmed our findings and reported that severe dry eye symptoms were associated with the female sex (p < 0.01) [23]. Sex hormones, particularly androgens, which are critical in tear function, could be the source of this feminine bias [24]. This could be explained by the insufficient tear secretion caused by estrogen shortage in menopausal women even though some research findings have shown that women on hormone replacement therapy may have an increased risk of dry eye [25].

Furthermore, we discovered that participants who use cell phones experienced moderate sleep-wake issues compared to others. Several pieces of research have already reported the impact of Internet, mobile phone, and screen usage on sleep quality and length. A study that was carried out in the United Kingdom found a link between nocturnal mobile phones and television use in increasing the risk of insufficient sleep duration among school-aged adolescents, and this association is much stronger when mobile phones are used in the dark [26]. In our study, there were significant associations between time spent on devices in studying and watching movies, sleep-cycle problems, eye tiredness, and eye dryness. These findings were similar to the results of a previous study that was conducted in Spain [19].

The overall sleep-wake score was 34.2 ± 8.8, with severe sleep-wake issues (67.2%) leading the way, followed by moderate sleep-wake difficulties (11.6%). However, regarding eye dryness, the overall score was 7.2 ± 5.9 with the most common groups experiencing mild to moderate symptoms (41.7%) and no symptoms (46.2%). A previous study in Saudi Arabia found that 71.4% of the subjects had severe eye dryness, 15% had moderate eye dryness, and 13.7% had mild eye dryness. However, 40.6% of participants mentioned they had never been diagnosed with dry eye disease, and 34.6% stated they had never experienced ocular dryness illness symptoms. The average overall Pittsburgh Sleep Quality Index (PSQI) score was 8.63, which was higher than our findings [7]. Although our study covered a large sample size, the study had some limitations. This was a cross-sectional study based on a self-administered questionnaire, which could have produced biases due to answers misinterpretation. Another limitation of the current study was the lack of information on the usage of hormone replacement therapy among females as well as the usage of drugs in systemic disorders such as hypertension. Therefore, prospective studies are recommended in the future and further action must be taken to raise awareness among college students regarding excessive screen time and its relation to dry eye symptoms and sleep-cycle problems.

## Conclusions

Our study concluded that university students in Saudi Arabia had significant sleep-cycle difficulties and mild to moderate eye dryness symptoms. Age, female gender, sleep duration, educational level, monthly income, and the increased time spent on screen were found to be associated with sleep-cycle problems and eye dryness symptoms.
